# The Information and Communication Technology Maturity Assessment at Primary Health Care Services Across 9 Provinces in Indonesia: Evaluation Study

**DOI:** 10.2196/55959

**Published:** 2024-07-18

**Authors:** Dewi Nur Aisyah, Agus Heri Setiawan, Alfiano Fawwaz Lokopessy, Nadia Faradiba, Setiaji Setiaji, Logan Manikam, Zisis Kozlakidis

**Affiliations:** 1 Department of Epidemiology and Public Health, Institute of Epidemiology and Health Care, University College London, London, United Kingdom; 2 Digital Transformation Office, Ministry of Health Republic of Indonesia, Jakarta, Indonesia; 3 Aceso Global Health Consultants Pte Limited, Singapore, Singapore; 4 International Agency for Research on Cancer, World Health Organization, Lyon, France

**Keywords:** public health centers, Puskesmas, digital maturity, infrastructure, primary health care, district health office, primary care clinics, Asia, Asian, Indonesia, ICT, information and communication technologies, information and communication technology, maturity, adoption, readiness, implementation, eHealth, telehealth, telemedicine, cross sectional, survey, surveys, questionnaire, questionnaires, primary care

## Abstract

**Background:**

Indonesia has rapidly embraced digital health, particularly during the COVID-19 pandemic, with over 15 million daily health application users. To advance its digital health vision, the government is prioritizing the development of health data and application systems into an integrated health care technology ecosystem. This initiative involves all levels of health care, from primary to tertiary, across all provinces. In particular, it aims to enhance primary health care services (as the main interface with the general population) and contribute to Indonesia’s digital health transformation.

**Objective:**

This study assesses the information and communication technology (ICT) maturity in Indonesian health care services to advance digital health initiatives. ICT maturity assessment tools, specifically designed for middle-income countries, were used to evaluate digital health capabilities in 9 provinces across 5 Indonesian islands.

**Methods:**

A cross-sectional survey was conducted from February to March 2022, in 9 provinces across Indonesia, representing the country’s diverse conditions on its major islands. Respondents included staff from public health centers (*Puskesmas*), primary care clinics (*Klinik Pratama*), and district health offices (*Dinas Kesehatan Kabupaten*/*Kota*). The survey used adapted ICT maturity assessment questionnaires, covering human resources, software and system, hardware, and infrastructure. It was administered electronically and involved 121 public health centers, 49 primary care clinics, and 67 IT staff from district health offices. Focus group discussions were held to delve deeper into the assessment results and gain more descriptive insights.

**Results:**

In this study, 237 participants represented 3 distinct categories: 121 public health centers, 67 district health offices, and 49 primary clinics. These instances were selected from a sample of 9 of the 34 provinces in Indonesia. Collected data from interviews and focus group discussions were transformed into scores on a scale of 1 to 5, with 1 indicating low ICT readiness and 5 indicating high ICT readiness. On average, the breakdown of ICT maturity scores was as follows: 2.71 for human resources’ capability in ICT use and system management, 2.83 for software and information systems, 2.59 for hardware, and 2.84 for infrastructure, resulting in an overall average score of 2.74. According to the ICT maturity level pyramid, the ICT maturity of health care providers in Indonesia fell between the basic and good levels. The need to pursue best practices also emerged strongly. Further analysis of the ICT maturity scores, when examined by province, revealed regional variations.

**Conclusions:**

The maturity of ICT use is influenced by several critical components. Enhancing human resources, ensuring infrastructure, the availability of supportive hardware, and optimizing information systems are imperative to attain ICT maturity in health care services. In the context of ICT maturity assessment, significant score variations were observed across health care levels in the 9 provinces, underscoring the diversity in ICT readiness and the need for regionally customized follow-up actions.

## Introduction

Digital health plays a significant role in enhancing health care services for people and has developed beyond providing electronic health records to supporting health care services provision, health surveillance, health literature, health research, and data-driven health policies [1-4]. The massive growth of technology use in health propelled to the top of the global agenda, as summarized in the World Health Organization’s (WHO) Global Strategy on Digital Health 2020‐2025 [1]. According to this report, each country is expected to adopt the strategies best suited to its conditions, culture, and values to reach its digital health sustainability.

In 2022, the Ministry of Health (MoH) of the Republic of Indonesia launched its health system transformation strategy. The transformation strategy had 6 pillars; one of them was primary health care transformation, which focused on strengthening promotive and preventive activities in its implementation to create more healthy people, improve health screening, and increase primary service capacity. Health care services in Indonesia are primarily provided at a public health care facility called *Puskesmas* (short for *Pusat Kesehatan Masyarakat* or public health centers) and community-based public health services (called *Posyandu*) since the 1980s.

*Puskesmas* in Indonesia are spread across all types of characteristic regions, such as urban, rural, remote, and very remote regions in Indonesia within all 38 provinces and 514 districts or cities. The number of *Puskesmas* has increased since 2017, from 9825 units to 10,374 in 2022. The ratio of *Puskesmas* in Indonesia to subdistricts was 1.4 in 2022. This illustrates that the ideal ratio of *Puskesmas* to subdistricts, namely a minimum of 1 *Puskesmas* in 1 subdistrict, has been fulfilled nationally. In terms of an average ratio, each *Puskesmas* serve 27,000‐30,000 residents in 2023.

*Puskesmas* serve two main functions: providing integrated individual health care services and essential public health services. Other tasks of *Puskesmas* are to provide continuous and comprehensive care, refer patients to specialists and hospital services, coordinate health services, and guide patients within the network of public health services. *Puskesmas* play roles in promotive, preventive, and curative services for the population, while primary clinics and other primary health services, such as private general practitioner (DPM) and private midwife (BPM), are more focused on curative and specific health services approaches. Other than that, *Puskesmas* also have a responsibility to provide technical guidance to primary clinics, DPMs, and BPMs as the networking partner institutions in the area. Besides *Puskesmas* and *Posyandu*, essential health services are provided by other primary care centers, such as clinics, DPMs, and BPMs. In 2023, the primary care clinics also increasingly grew and reached more than 11,000 units, while DPM and BPMs reached 5800 units across all 38 provinces in Indonesia.

In terms of organizational governance, *Puskesmas* are considered the technical implementation unit of district health offices. In this case, the district health office is responsible for providing guidance, monitoring, and evaluation for *Puskesmas* in its region. However, *Puskesmas* have autonomy to synchronize and harmonize the health development goals in their working area. All health service activities in the area are conducted by *Puskesmas* and their networking partners (ie, primary clinics, DPMs, and BPMs). *Puskesmas* coordinate and report these activities to the district health office on an annual, monthly, and weekly basis using either electronic or nonelectronic systems. The reports include (1) activity report, (2) financial report, (3) field survey report, (4) related cross-sector reports, and (5) the health services network (clinics, DPM, BPM) reports in the area. This reporting scheme continues by the district health office to the province health office and then to the MoH as the national authority.

The health services and public health programs that were routinely reported using an electronic information system included maternal and child health, nutrition, surveillance, as well as disease prevention and control. These programs use multiple, separate applications that currently lack interoperability data standards. Besides that, there are multiple electronic medical record systems widely developed by hundreds of private vendors, designed to provide patient medical records for health care providers. These systems include *Sistem Informasi Puskesmas* (SIMPUS) for *Puskesmas* and *Sistem Informasi Klinik* (SIMKLINIK; clinic information systems) for primary clinics, as well as telemedicine and tele consultation platforms. These syetms are still not integrated and interoperable with other public health information systems developed by the MoH.

Indonesia has pursued continuous improvements in the national digital health implementation. To this end, the COVID-19 pandemic accelerated the introduction of national digital health capacity and raised the number of daily health applications users, currently exceeding 15 million people [5,6]. Through the launch of the Blueprint of Digital Health Transformation Strategy 2024, the government committed to using digital technology and data for public health to support the realization of a healthy Indonesia. The priority of Indonesia’s digital health transformation activities include the integration and development of health data, the integration and development of application systems, and the developmet of a health technology ecosystem. These transformations aimed to collect standardized health data into a centralized platform named *Satu Sehat* (One Health Data). This process began with designing the health data architecture and interoperability as well as assessing the current infrastructure and security levels [7].

Implementing such a large-scale digital transformation requires technical maturity in the health sectors’ information and communication technology (ICT). The critical factors of ICT maturity include infrastructure, policies, human capital development, change management, strategy, leadership, partnership, and collaboration [8]. In 2017, the MoH used the Health Metrics Network of the World Health Organization as the basis for a national digital health strategic framework, incorporating a holistic approach in planning, developing, implementing, and evaluating the use of ICT in health services. The strategic framework contained seven components: (1) governance and leadership; (2) strategy and investment; (3) services and application; (4) standards and interoperability; (5) infrastructure; (6) legislation, policy, and compliance; as well as (7) workforce [2,9].

Chanyagorn and Kungwannarongkun [10] developed an ICT maturity assessment tool to explore ICT readiness in small- and medium-sized organizations in middle-income countries, covering both public and private sectors. This assessment tool was used to evaluate the ability of consumers, businesses, and governments to use ICT to their advantage; however, this study assessed ICT maturity in sample governmental public health services’ facilities and institutions. The most significant aspect of this tool was the assessment of maturity for various aspects, as it could be adjusted to reflect best the different mixes of conditions in middle-income countries like Indonesia. The tool assessed four main ICT factors: (1) infrastructure, (2) hardware, (3) software and system information, as well as (4) people and human resources [10].

Therefore, as part of the digital health development in Indonesia, it is important to understand the ICT maturity level in services immediately after the pandemic, as a benchmark for future ICT initiatives. This study focused its assessment on ICT maturity across primary health care services and district health offices in Indonesia, applying the ICT maturity assessment tool for middle-income countries. To maintain a high level of inclusivity of services across different geographical regions, the evaluation of digital health capability was investigated in 9 provinces, spread over the 5 biggest islands in Indonesia. This study is the first study to assess the ICT maturity through middle-income country approaches for public health centers, primary clinics, and district health offices.

## Methods

### Data Collection

A cross-sectional survey design involving the ICT maturity assessment tool for middle-income countries was used in 9 provinces in Indonesia. Targeted stratified sampling was applied to choose the participants from provinces on the 5 largest islands in the country. DKI Jakarta, West Java, Banten, and East Java provinces on Java Island; Aceh province on Sumatra Island; East Kalimantan province on Kalimantan Island; Central Sulawesi province on Sulawesi Island; West Nusa Tenggara on the Nusa Tenggara Islands; and Maluku province on Maluku and Papua Islands. Provinces chosen on the 5 largest islands were based on region representativeness and health facility characteristics, which included urban, rural, and remote areas.

The targeted respondents were representatives of 3 main health sites in the province, including public health centers (*Puskesmas*), primary care clinics (*Klinik Pratama*), and district health offices (*Dinas Kesehatan Kabupaten*/*Kota*). A letter of invitation was sent to the targeted province district health office as the official invitation for an electronic survey and a forum group discussion (FGD) session. Each participant involved in the survey and FGD session was selected based on top management’s assignment to attend the electronic survey and FGD meeting. To address participant selection and response biases, all assigned participants from all regions needed to fulfill the following inclusion criteria: (1) personnel who handled and oversaw the health information system implementation in each site, (2) with knowledge of the health information system used at the health site, and (3) with the ability to communicate with local staff to complete the survey accurately.

**Figure 1.**
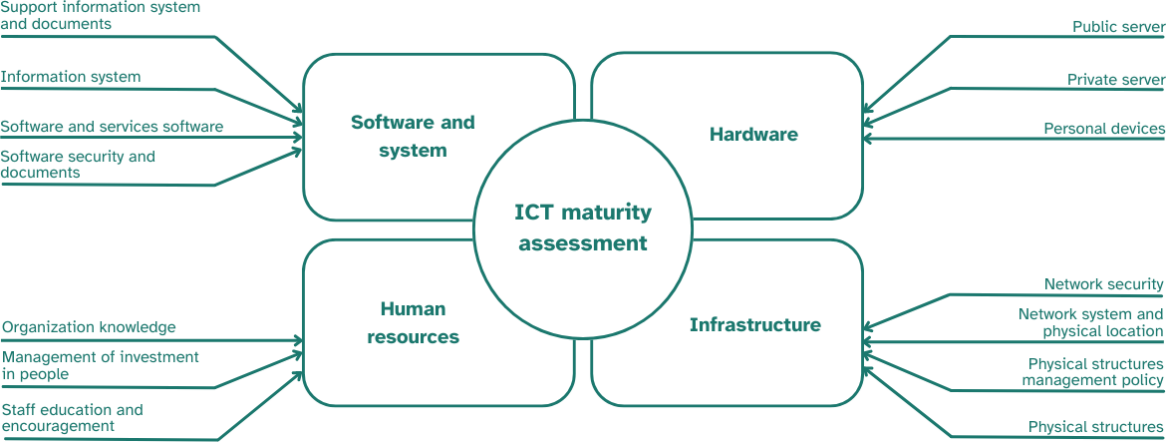
The modified components evaluated in the information and communication technology (ICT) maturity assessment.

An ICT maturity assessment questionnaire was adopted and modified, adjusting to the local country’s situation (Multimedia Appendix 1). The modification was made to the questions of each ICT subcomponent, especially the organization knowledge component, software security and document component, and network security component (Figure 1). A pilot survey test was conducted with 20 participants to gather feedback from participants, ensuring the questions were easy to understand, determining the time required to complete the questionnaire, and capturing suggestions to improve the assessment form. The questionnaire (Multimedia Appendix 2) consisted of four sections: (1)human resources, covering the availability and capacity of the personnel in using ICT; (2) software and systems, covering the number of health information systems used, data reporting in the health information system, troubleshooting, and maintenance performance; (3) hardware, including the availability of PCs/laptops, servers, storage, and manual entry data; and (4) infrastructure, including access to the internet access, electricity, and physical facility/building (Figure 1).

The survey was conducted by gathering the respondents through web-based meetings. The process was completed between February and March 2022. There were 237 respondents divided into 7 FGDs, with each FGD facilitated by 5 facilitators. The data were collected through 2 sessions in the meetings, which took 2.5 hours to complete. The 2 sessions were as follows: (1) filling out the questionnaires for the quantitative score and (2) an FGD session to discuss the survey findings and further explore the assessment results obtained. Each individual component of the ICT maturity questionnaire was discussed in more detail to obtain more comprehensive qualitative information. Discussions were continued until thematic saturation was reached.

### Data Analysis

**Figure 2.**
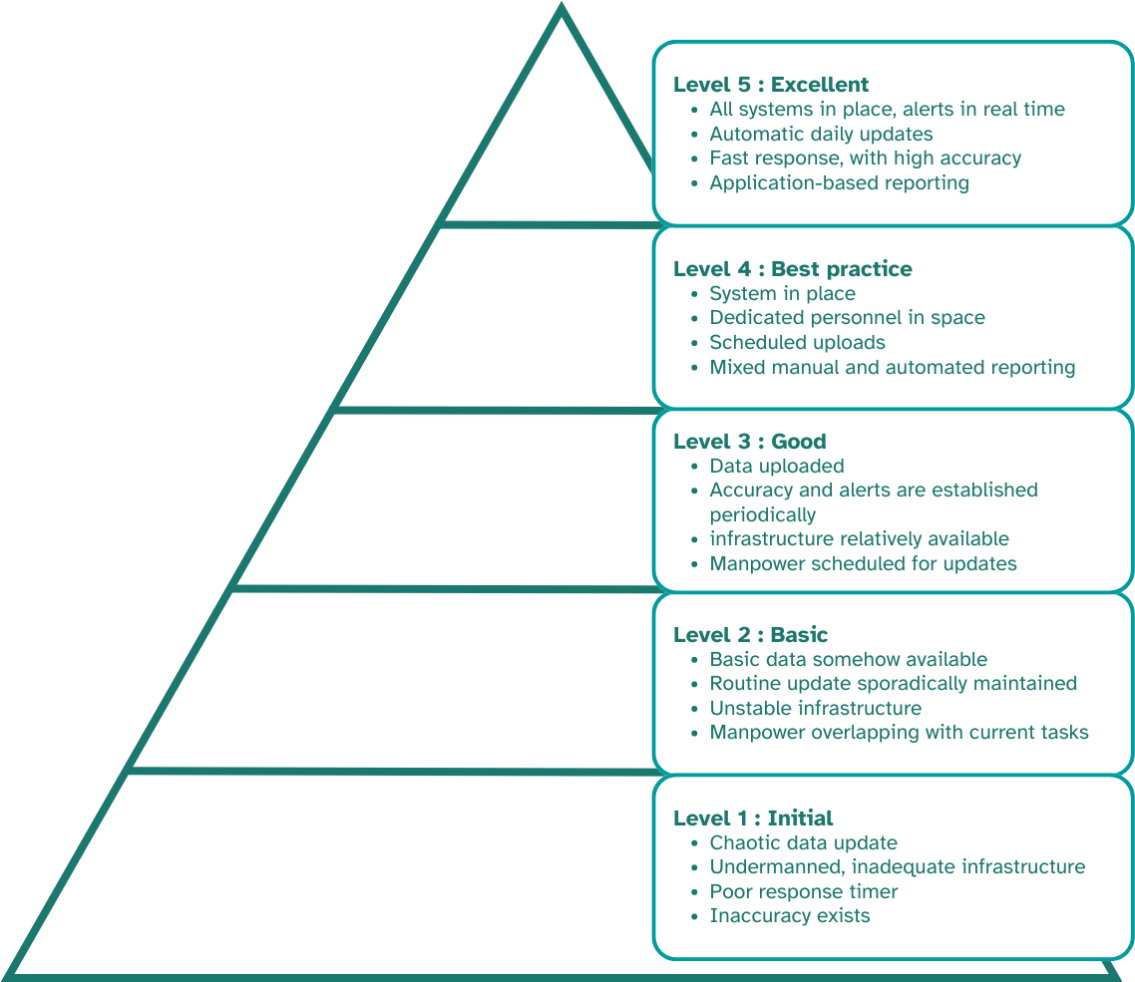
Information and communication technology maturity level.

The ICT maturity assessment tool consisted of 14 subsections containing 30 questions (Multimedia Appendix 2). The participants’ responses to these questions were categorized into 5 levels of digital maturity: (1) initial, (2) basic, (3) good, (4) best practice, and (5) excellent (Figure 2), with a score of 1 indicating the lowest level of digital maturity (initial) and 5 representing the highest (excellent). The electronic survey responses were recorded by our team using the Mentimeter forms database tools, and the FGD results were input into a table using Microsoft Excel. The scores of each participating organization, including public health centers, primary care clinics, and district health offices, were used to determine their ICT maturity levels. The organization’s score was calculated and averaged for each province.

The analysis was performed in Microsoft Excel. The scores from the 4 components were used to produce the mean score. A comparison analysis at the district and province levels was also generated. The FGD, held via Zoom, was recorded, and qualitative information obtained from participants was categorized based on the ICT component to be summarized as the FGD results for all provinces.

### Ethical Considerations

According to the “Exemption” section of the Indonesian MoH’s National Guidelines and Standards for Ethical Research and Development in Health (2017), studies are exempted from review process if there is no/little potential risk/harm arising from the conduct of the research or when the information collected is available from the public domain; if they involve the use of educational tests (cognitive, diagnostic, attitude, and achievement); and if they involve survey or interview procedures, or public behavior observations. Research and demonstrations conducted by or subject to the approval of a department or agency and designed to study, evaluate, or assess the benefits of public programs or services, as well as other goods and services identified in the regulations, are also exempted from obtaining ethics approval. The original guidelines are available in Indonesian language of the MoH’s guidelines (Chapter IIIB, point 2) [11]. The participants’ data were also anonymized, following the Indonesian MoH’s National Guidelines and Standards for Ethical Research and Development in Health (2017), Chapter IIIB, point 2.

## Results

The study respondents were 237 in total and included 121 representatives of public health centers, 67 IT staff from district health offices, and 49 primary care clinic staff (Table 1). The provinces of East Java, Aceh, East Kalimantan, and West Nusa Tenggara had the largest representation among the participating public health centers, with 27, 17, 15, and 15 public health centers from each province, respectively. As for the district health offices, Aceh, West Nusa Tenggara, and East Kalimantan each had 11, 9, and 9 district health offices, respectively, while East Java had the highest representation with 13 district health offices. Notably, representatives from West Nusa Tenggara led the primary care clinic segment, accounting for 12 primary care clinics, followed by East Java with 9 primary care clinics and Aceh with 8 primary care clinics.

**Figure 3.**
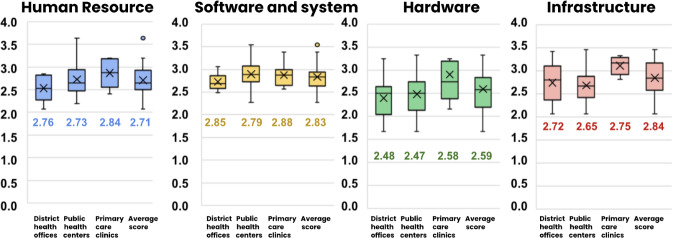
Information and communication technology maturity scores across four main components at each site.

On average, the ICT maturity scores were broken down as follows: 2.71 for human resource, 2.83 for software and systems, 2.59 for hardware, and 2.84 for infrastructure, resulting in an overall average score of 2.74 (Figure 3). Based on the analysis, health care providers in Indonesia had an ICT maturity between basic and good according to the ICT maturity level pyramid.

**Table 1.** The study participants.

Island and province	Public health center, n	District health office, n	Primary care clinic, n	Subtotal, n
Java, DKI Jakarta	6	5	1	12
Java, West Java	13	6	5	24
Java, Banten	5	2	1	8
Sumatra, Aceh	17	11	8	36
Java, East Java	27	13	9	49
Nusa Tenggara, West Nusa Tenggara	15	9	12	36
Kalimantan, East Kalimantan	15	9	6	30
Sulawesi, Central Sulawesi	11	8	2	21
Maluku, Maluku	12	4	5	21
Total	121	67	49	237

**Figure 4.**
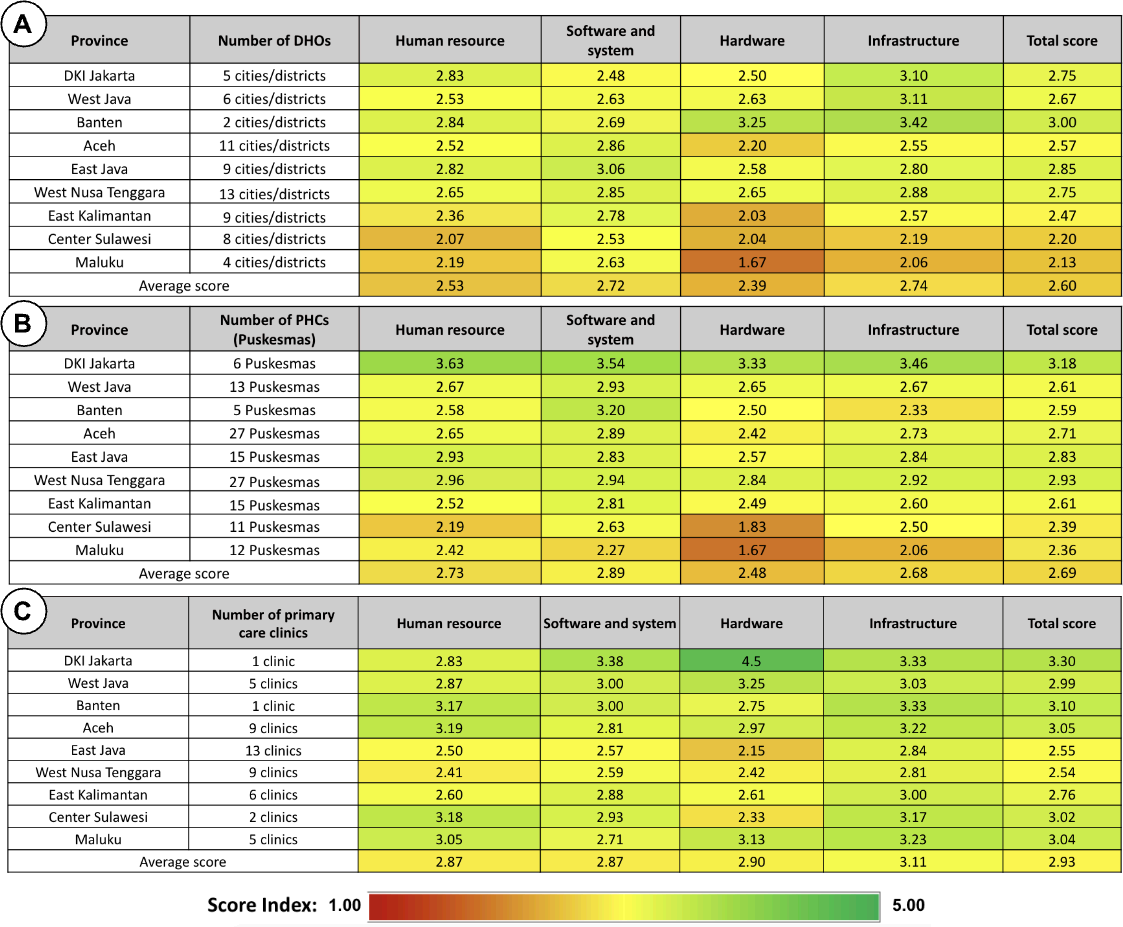
Average scores for the information and communication technology maturity level at (A) district health offices (DHOs); (B) public health centers (PHCs); and (C) primary care clinics.

There were variations in the ICT maturity scores by province (Figure 4). On average, the ICT maturity score at the district health offices level was 2.60. Banten Province had the best overall score (3.00), followed by East Java (2.85), DKI Jakarta (2.75), and West Nusa Tenggara (2.75). At the public health center level, the average score was 2.69, with the highest average score belonging to Jakarta (3.18), followed by West Nusa Tenggara (2.93) and East Java (2.83). The average ICT maturity scores at the primary care clinics level were 2.87 for human resource capability and system management, 2.87 for software and information systems, 2.90 for hardware, and 3.11 for infrastructure. The DKI Jakarta province had the highest average ICT maturity score at the primary care clinic level (3.30), followed by Banten (3.10) and Aceh (3.05).

**Figure 5.**
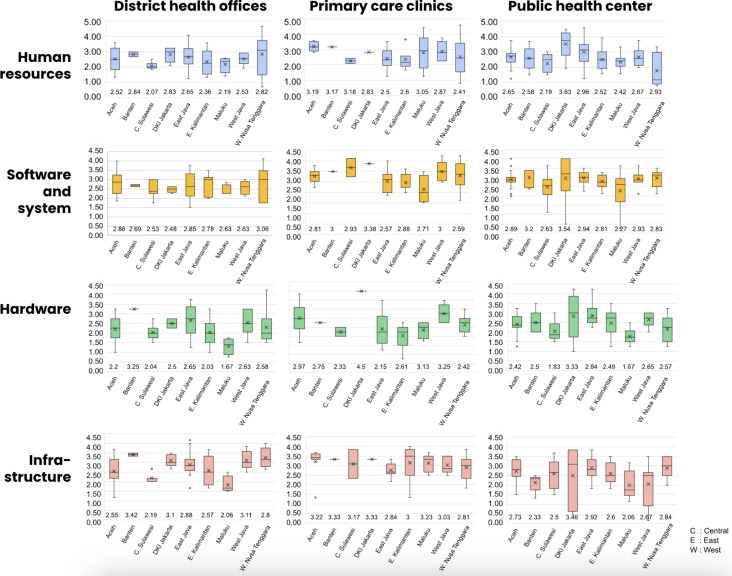
Information and communication technology maturity assessment score ranges across various provinces and health care levels.

Our analysis revealed that the public health centers, primary care clinics, and district health offices exhibited varying degrees of maturity across provinces (Figure 5). Important findings included the human resource scores, where Aceh, Banten, and Sulawesi had better developed human resource capacities, with ratings exceeding 3. A total of 9 provinces had scores ranging from 2 to 3 when it came to software and system maturity; the only exceptions were the public health centers and primary care clinics in DKI Jakarta, with scores of 3.54 and 3.38, respectively. In terms of hardware maturity, the majority of regions scored between 2 and 3, although some had significantly lower scores than others, such as Maluku’s district health office (1.67) and the *Puskesmas* in Central Sulawesi (1.83). Primary care clinics typically showed greater maturity in terms of infrastructure, with an average score of 3 or higher. However, Maluku’s public health center received a score of 2.06, whereas the district health offices in Maluku and Central Sulawesi received a lower score, ranging around 2.

**Figure 6.**
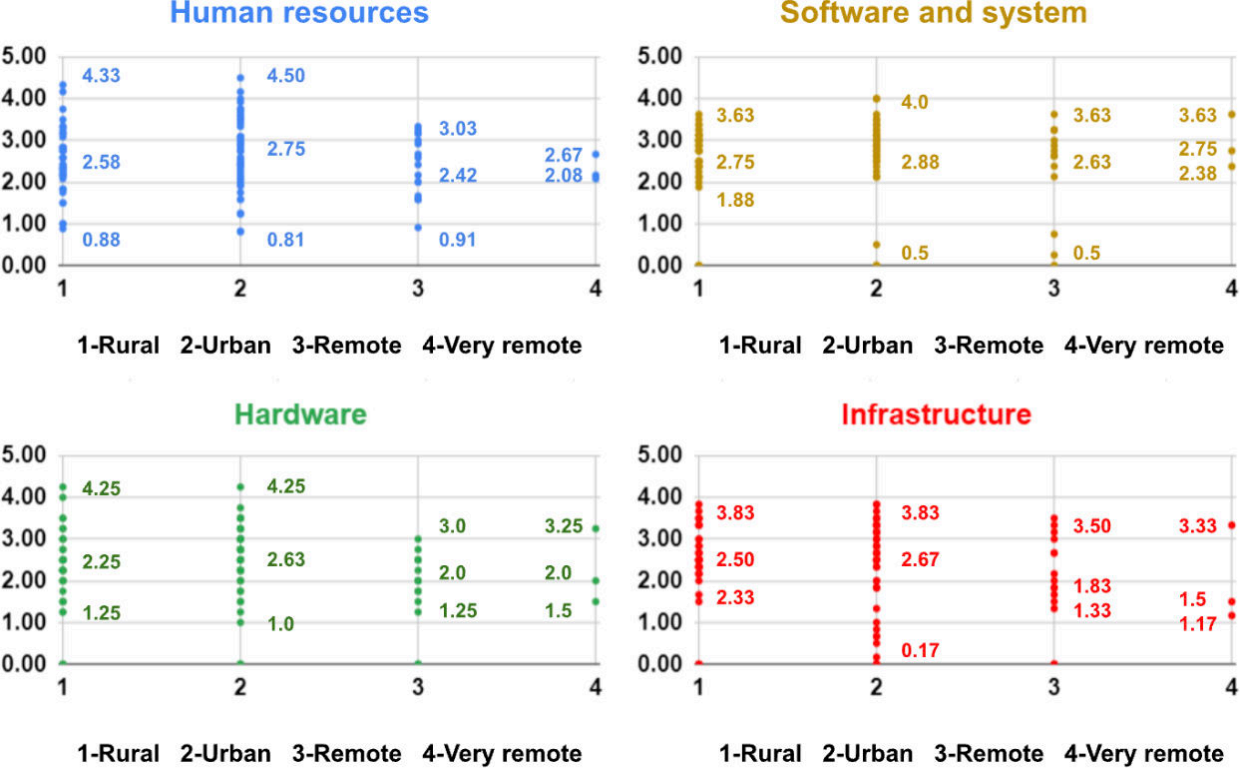
Information and communication technology maturity scores scatter plot by main components and site locations in the 9 targeted provinces.

When examining the differences in ICT maturity based on the location of public health centers, the distribution is evident. Figure 6 shows a detailed scatter plot graph created with the ICT maturity assessment scores, indicating differential maturity levels across urban and remote locations. In terms of human resources, it is apparent that isolated locations have lower maturity scores compared to their equivalent urban sites, although urban and rural areas have different variances. In addition, there are significant differences in the software maturity scores between rural and urban areas. The software maturity varies in rural areas compared to urban areas, where the scores are constantly higher. On the other hand, software maturity varies in remote places.

The lower scores for the software system maturity indicated that the facility’s questionnaire responses reflected qualities that hindered digital transformation or digital maturity. Key issues identified included the absence of trained or specialized personnel for data input and IT. Additionally, there was an excessive number of applications or information systems mandated by central or local governments. Despite the abundance of applications, not all were used effectively, and many lacked interoperability, resulting in data duplication and increased workloads for health workers. Hardware components were scored based on several factors, including the reliance on manual, paper-based methods or computer-based Excel tables for data input; the insufficient number of personal computers available for health care services and data input; the type of data storage used, such as computer-based or cloud-based to minimize data loss; and the quality and stability of the data server. For infrastructure, the scores for some regions were lower due to the poor internet quality in health facilities and the inconsistent power availability, which was not guaranteed 24 hours a day. In some cases, power outages occured even during operational hours.

Regional differences in hardware quality were also evident, with varying degrees of quality found in rural, urban, and remote locations. While remote areas had challenges due to lower quality or limits, rural and urban areas generally had higher standards for hardware quality. The trends seen in hardware and infrastructure were strikingly comparable, highlighting a shared tendency between these elements. These findings offer important insights into the particular opportunities and problems in various situations and they portrait an intricate picture of ICT maturity across various geographies. Additionally, these findings highlight specific area characteristics, which may aid the government in deciding appropriate approaches for ensuring the effective implementation of digital health services in these areas.

In addition to assessing digital maturity through scoring, we conducted FGDs, the findings of which highlighted a number of staffing- and human resources–related issues. First, there were people in the health care facilities and district health offices with a variety of backgrounds, but not all of them were IT literate. Second, data input was typically done by health officials or health workers; these staff members lacked professional IT certification or training. Third, data analysis was usually performed by nurses and staff at health centers due to a shortage of professionals with necessary capabilities. Fourth, a large number of different unintegrated health information systems added to the burden of data reporting. Lastly, the lack of hiring civil servants to fill the paucity of IT professionals with computer knowledge was a further obstacle.

In terms of software and information systems, issues included frequent system disruptions, poor system performance, and operational difficulties. The largest issue with software was the sheer number of health information systems that required data input but were not interoperable. For instance, a question about immunization status appeared in two existing systems: the system for tracking toddlers’ nutritional status and the immunization system. As there were no data integration in place, health care personnel were forced to continually enter the same data into multiple systems as a result of this redundancy.

The complexity was further increased by the ongoing addition of new applications from numerous sources, such as the MoH, other governmental organizations, or municipal governments. These applications frequently demanded a lot of data entry, which made data management quite difficult. Furthermore, the prevalence of paper-based reporting techniques persisted even in the face of digital alternatives. These difficulties highlight the need for more efficient and integrated solutions in the health care industry by impeding the overall simplification of data entry and system operations.

Numerous hardware-related issues emerged during the FGDs. Program managers frequently had to use their own laptops because their public health centers did not have enough resources. Despite the fact that the office had multiple computers, they were for shared use and in some subdistricts, the computers were limited and used only for the registration and administrative sections. In others, there were only 2-5 computers available to fulfill the management requirements of the entire public health center. Staff members occasionally used their personal laptops for data reporting. Furthermore, there were service recording delays due to PC storage limits. In addition, operational difficulties were exacerbated when individuals’ PCs experienced slowness or malfunctions and when data storage was not centralized but rather restricted to individual PCs without cloud backup.

Regarding infrastructure, the intermittently weak signal on Wi-Fi and internet-connected devices in several places was mentioned. The employees routinely used Wi-Fi, but they frequently ran into network issues. Consequently, all reporting needed to be completed manually in certain locations or by using personal mobile data or paper-based data collection. Moreover, server outages occasionally caused delays or even entirely stopped the data recording process. Additionally, there was no dedicated data storage, thus data were at risk during sporadic power outages, which were particularly problematic in situations such as wildfires.

## Discussion

### Principal Findings

The analysis of ICT maturity in Indonesia’s care providers reveals an overall average score of 2.74, indicating maturity between basic and good levels, aligned with the ICT maturity level pyramid. Variations exist across provinces and health care levels, with Banten Province exhibiting the highest overall score. The DKI Jakarta Province stands out with superior scores at the public health center and primary care clinic levels. Disparities in human resource, software, hardware, and infrastructure maturity exist between provinces and health care levels, with rural areas generally lagging behind urban areas. These findings underscore the importance of tailored strategies to address regional disparities and enhance digital health service implementation effectively.

The ICT maturity level has the potential to affect the quality of national health services, with the highest impact felt in middle-income countries. Excellent ICT maturity can lead to a good digital health implementation and potentially contribute to the improvement of health care, such as quality of care, supplies and logistics, training and communication, community engagement and participation in health services, as well as the availability and use of routine data by decision-makers [12-14]. The current research provides a snapshot of the ICT maturity in several health care services across Indonesia and explores factors that would require further attention.

The results of the questionnaires show the variety of ICT maturity among provinces, districts, and health facilities, with variations in critical components, such as human resource capabilities, software and information systems, hardware, and infrastructure. These disparities not only indicate the different provinces’ ICT readiness but also highlight potential areas for improvement and development to enhance health care service delivery and information management in each respective region. Understanding these variations is crucial for devising targeted strategies to address specific ICT-related challenges and opportunities within the health care system across diverse geographical locations.

The development of ICT in Indonesia is facing tough challenges due to the country’s geography, consisting of thousands of islands and many cultures, affecting educational, social, and economical aspects in the country. Moreover, Indonesia as an archipelago has many remote areas where telecommunication and internet service providers cannot develop the sufficient infrastructure; as such, these areas are unavoidably underrepresented [14-16]. Indonesia ranked 111 on the 2017 ICT Development Index, falling behind other countries in Southeast Asia like Singapore (ranking 18), Brunei Darussalam (ranking 53), Malaysia (ranking 63), Thailand (ranking 78), and Philippines (ranking 101) [17].

Regarding human resources, often staff members do not have adequate digital literacy, and yet are required to report using IT processes, as it was also indicated in our work [18,19]. Moreover, there is a shortage of staff with computer expertise and insufficient data management training opportunities to mitigate this issue. The same is described in Malaysia, where challenges related to human resources include workload, readiness, skills, and user dependency. Additionally, tasks often require health workers to focus on mining data, instead of improving service provision [20-23].

Indonesia also faces a mind shift challenge for staff, as the plurality of overlapping systems comes against a context of well-established manual data collection, using paper or status books. In contrast, frequent input of overlapping variables across multiple software, adds to the work burden of individual health care staff and makes the digital health process inefficient. Countries in sub-Saharan Africa have also faced a similar challenge: they documented 738 distinct digital health interventions at different levels of functioning in the sub-Saharan African region over the past 10 years. One in 5 of those did not have a link to any health service outcomes, and only half could be classified as “established” at the end of the study period. Two of every 3 were focused solely on solutions for a single health care activity, limiting integration [20]. This aspect has not been researched as yet for the countries neighboring Indonesia.

The existence of infrastructure remains the main challenge in developing and implementing e-governments in middle-income countries [24,25]. In the health care industry, the continuity of using ICT in daily and routine operations depends on the availability of a robust IT infrastructure. Unfortunately, many middle-income countries lack the necessary infrastructure to support digital health, specifically telecommunication and electricity networks coverage [25-27]. This is also supported by other studies that found that IT systems used in the health care industry would only be optimal, effective, and efficient when adequate facilities and infrastructure supported them [28-31].

The discrepancies in infrastructure availability across the provinces in Indonesia directly affect a stable internet connection. Frequent internet signal downtime, network problems, poor connection, and insufficient computer availability, including the lack of electricity supplies in some remote areas, were found in this study, showing the need for further improvements in technology infrastructure and facilities. This lack and instability of IT infrastructure, such as the limitation of internet access, electricity supply, and availability of computers, is common across low- and middle-income countries, as similar findings were described for Brazil, sub-Saharan Africa as a whole, Sierra Leone, and Tanzania [32-36].

These findings are crucial for gaining a better understanding to support the Indonesian health technology transformation mentioned in the Blueprint of Health Transformation Strategy 2024. The foundation to transform the health systems lies in having a solid platform architecture design and infrastructure to implement integrated and interoperable health systems. The blueprint highlighted the plan to integrate all electronic medical record systems from public health centers, hospitals, primary care clinics, and laboratories into the *Satu Sehat* platform and to adopt the *Satu Sehat* standard. This study showed that to support the implementation of digital health transformation, the government should identify the gap; map the area based on capacity; and provide assistance to improve the software, hardware, infrastructure, and human resource capacity, especially in areas with lower ICT maturity scores.

### Strengths and Limitations

The strengths of the study involve exploring ICT maturity in Indonesia, engaging several health care service sites (ie, *Puskesmas*, clinics, and health departments). Furthermore, the results of the FGDs provided a more detailed overview of the challenges related to the four assessed components, thus highlighting areas for future improvements to support the ongoing digital transformation of health care.

The limitations of the study are as follows: (1) the involvement of public health centers and primary clinics in the study was limited in each province and perhaps not entirely representative, although health departments involved in the study represented 80%‐90% of the 9 targeted provinces and districts; (2) other health care facilities, such as hospitals, laboratories, and pharmacies, were not involved in this study, and as such, additional aspects of needs or challenges may exist that have not been highlighted as yet; this may have implications for the representativeness of health care data users by volume; (3) the representativeness based on infrastructure distribution and regional characteristics (eg, urban, rural, remote, and very remote regions) should be viewed only as indicative, as health care services from more remote rural regions are less likely to have access to stable internet connection, and thus, unable to complete such questionnaires distributed by digital channels; (4) the respondents’ capacity can influence data completeness, and thus, it will be useful for future iterations if staff representing more functions of the health care centers are able to complete the questionnaire.

### Conclusions

This study investigated for the first time the variations of ICT maturity across the health care systems (eg, public health centers, primary clinics, and district health offices) in 9 provinces in Indonesia, underscoring the diversity in ICT implementation and readiness. The maturity of ICT use was influenced by several critical components, specifically enhancing human resources, ensuring infrastructure, the availability of supportive hardware, and optimizing information systems. The findings of this study are in line with similar studies in other middle-income countries in the world. Our results demonstrate that to attain ICT maturity in health care services in Indonesia, it is imperative to address all of the above aspects, as each represents ongoing needs and has been shown to be equally important and necessary in the field.

## Supplementary material

10.2196/55959 Multimedia Appendix 1 Modified information and communication technology (ICT) maturity assessment.

10.2196/55959 Multimedia Appendix 2 Questionnaire and in-depth interview guidelines.

## Abbreviations

BPM: private midwife
DPM: private general practitioner
FGD: forum group discussion
ICT: information and communications technology
MoH: Ministry of Health
WHO: World Health Organization

## References

[1] (2021). Global strategy on digital health 2020-2025. World Health Organization.

[2] Woods L, Eden R, Pearce A (2022). Evaluating digital health capability at scale using the digital health indicator. Appl Clin Inform.

[3] Abernethy A, Adams L, Barrett M (2022). The promise of digital health: then, now, and the future. NAM Perspect.

[4] (2017). ICT trends. Asian and Pacific Training Centre for Information and Communication Technology for Development (APCICT).

[5] Pitaloka AA, Nugroho AP (2021). Digital transformation in Indonesia health care services: social, ethical and legal issues. J STI Policy Manage.

[6] Saputra YE, Worsito SB, Firdaus DS, Listiyandini RA (2022). Indonesia Post-Pandemic Outlook: Rethinking Health and Economics Post-COVID-19.

[7] (2021). Blueprint of Digital Health Transformation Strategy 2024. Indonesia Ministry of Health.

[8] Zaeid ANH, Khairalla FA, Al-Rashed W, Zaied H (2007). Assessing e-readiness in the Arab countries: perceptions towards ICT environment in public organisations in the state of Kuwait. Electro J e-Govern.

[9] (2017). Strategi e-kesehatan nasional. Indonesia Ministry of Health.

[10] Chanyagorn P, Kungwannarongkun B (2011). ICT readiness assessment model for public and private organizations in developing country. IJIET.

[11] Pedoman dan standar etik penelitian dan pengembangan kesehatan nasional [Article in Indonesian]. Indonesia Ministry of Health.

[12] (2018). UNICEF’s approach to digital health. UNICEF Health Section Implementation Research and Delivery Science Unit and the Office of Innovation Global Innovation Centre.

[13] Shaygan A, Daim T (2023). Technology management maturity assessment model in healthcare research centers. Technovation.

[14] Sari EN (2022). Navigating the landscape of digital health landscape, Indonesia. Health Intervention and Technology Assessment Program (HITAP) Ministry of Public Health (MoPH) Thailand.

[15] Amin M (2018). ICT for rural area development in Indonesia: a literature review. JITU.

[16] Handayani PW, Yazid S, Bressan S, Sampe AF (2020). Information and communication technology recommendations for the further development of a robust national electronic health strategy for epidemics and pandemics. J Sistem Inf (J Inf Sys).

[17] (2017). Measuring the information society report: volume 1. International Telecommunication Union.

[18] (2019). Empowering the health workforce: strategies to make the most of the digital revolution. OECD.

[19] Karamagi HC, Muneene D, Droti B (2022). eHealth or e-chaos: the use of digital health interventions for health systems strengthening in sub-Saharan Africa over the last 10 years: a scoping review. J Glob Health.

[20] Ajadi S (2020). Digital health: a health system strengthening tool for developing countries. GSM Association.

[21] (2017). Strategy on human resources for universal access to health and universal health coverage. PAHO.

[22] ChePa N, Md Jasin N, Abu Bakar NA (2018). Information system implementation failure in Malaysian government hospitals: how change management helps?. J Telecommun Electron Comput Eng.

[23] Myyrä N (2016). Digital health interventions for employees: are digital health interventions able to improve a company’s performance?. Helsinki Metropolia University of Applied Sciences.

[24] Ndou V (2004). E-government for developing countries: opportunities and challenges. E J Info Sys Dev Countries.

[25] Delponte L, Grigolini M, Moroni A, Vignetti S (2015). ICT in the developing world: in-depth analysis. Sci Technol Options Assess.

[26] Omotosho A, Ayegba P, Emuoyibofarhe J, Meinel C (2019). Current state of ICT in healthcare delivery in developing countries. Int J Onl Eng.

[27] Djawad YA, Suhaeb A, Mustakim R, Jaya H (2019). The development of an intelligent e-health mobile application in Indonesia: a preliminary study. INSIST.

[28] Ebnehoseini Z, Tabesh H, Deldar K, Mostafavi SM, Tara M (2019). Determining the hospital information system (HIS) success rate: development of a new instrument and case study. Open Access Maced J Med Sci.

[29] Afrizal SH, Handayani PW, Hidayanto AN, Eryando T, Budiharsana M, Martha E (2019). Barriers and challenges to primary health care information system (PHCIS) adoption from health management perspective: a qualitative study. Informatics in Medicine Unlocked.

[30] Health Metrics Network (2007). Framework and Standards for Country Health Information Systems.

[31] Sayyadi Tooranloo H, Sepideh S, Arezoo Sadat A (2021). Evaluation of failure causes in employing hospital information systems. J Syst Manag.

[32] Atashi A, Khajouei R, Azizi A, Dadashi A (2016). User interface problems of a nationwide inpatient information system: a heuristic evaluation. Appl Clin Inform.

[33] Yoshiura VT, de Azevedo-Marques JM, Rzewuska M (2017). A web-based information system for a regional public mental healthcare service network in Brazil. Int J Ment Health Syst.

[34] Odekunle FF, Odekunle RO, Shankar S (2017). Why sub-Saharan Africa lags in electronic health record adoption and possible strategies to increase its adoption in this region. Int J Health Sci (Qassim).

[35] Sukums F, Mensah N, Mpembeni R (2015). Promising adoption of an electronic clinical decision support system for antenatal and intrapartum care in rural primary healthcare facilities in sub-Saharan Africa: the QUALMAT experience. Int J Med Inform.

[36] Chukwu E, Garg L, Foday E, Konomanyi A, Wright R, Smart F (2022). Electricity, computing hardware, and internet infrastructures in health facilities in Sierra Leone: field mapping study. JMIR Med Inform.

