# Supplementary material for: The Information and Communication Technology Maturity Assessment at Primary Health Care Services Across 9 Provinces in Indonesia: Evaluation Study
Source: JMIR Med Inform. 2024 Jul 18;12:e55959. doi: 10.2196/55959 (PMC11269960; doi:10.2196/55959)
Supplement: Multimedia Appendix 1 [file medinform-v12-e55959-s001.docx]

**Appendix 2. Questionnaire difference**

**→** [Manuscript Draft_ICT Maturity Assessment_Suggestion](https://docs.google.com/document/d/1YKUx2zV7y6_UD8d04W1Ab1z7tYaaNj7zpORzXruNDU4/edit)

| Chanyagorn and Kungwannarongkun (2011) | 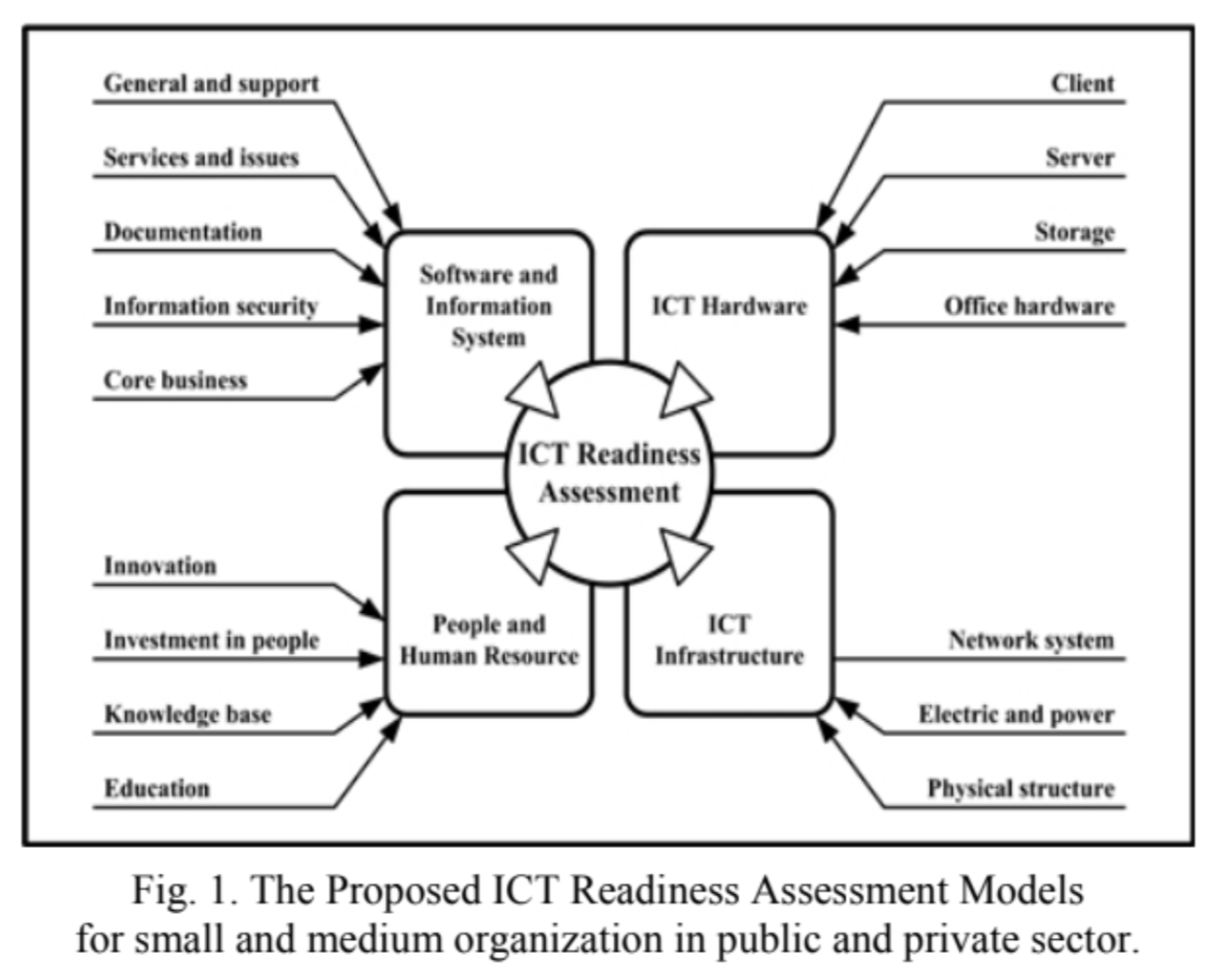 |
| --- | --- |
| Our modified version | 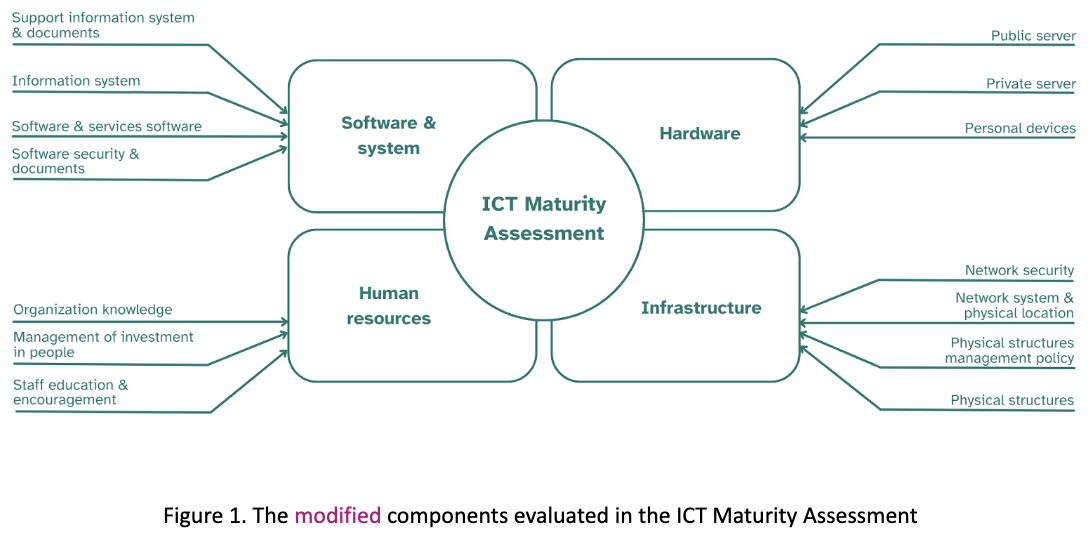 |

| **No** | **Chanyagorn and Kungwannarongkun (2011)** | **Our modified version** |
| --- | --- | --- |
| 1 | Top left section : Software and Information system   1. General and support 2. Services and issues 3. Documentation 4. Information security 5. Core business   *General software & information system pertaining governmental needs* | Top left section : Software and system   1. Support information system & documents 2. Information system 3. Software & services software 4. Software security & documents   *Our focus is on software & information systems in health services utilization, especially in the government's health community center (Puskesmas) → The questions include identifying Information Systems being used, Support for IS technicians, Number of IS and which ones that are Effectively used, Data completeness in each IS and Data duplication, Benefits of IS and user satisfaction* |
| 2 | Top right section : ICT Hardware   1. Client 2. Server 3. Storage 4. Office Hardware | Top right section : Hardware   1. Public server 2. Private server 3. Personal devices   *Ours focus on assessing hardwares needed for Puskesmas such as PC/Computer specifications, Manual/Paper-based data entry behavior, data server, data storage* |
| 3 | Bottom right section : Infrastructure   1. Network system 2. Electric and power 3. Physical structure | Bottom right section : Infrastructure   1. Network security 2. Network system & physical location 3. Physical structures management policy 4. Physical structures   *Our questionnaire covers topics such as facility’s internet quality and electricity condition in the vicinity* |
| 4 | Bottom left section : People and human resource   1. Innovation 2. Investment in people 3. Knowledge base 4. Education | Bottom left section : Human resource   1. Organization knowledge 2. Management of investment in people 3. Staff education & encouragement   *Ours include questions about data entry and data analyst personnel, whether Puskesmas have specified personnel’s skill requirement and training* |
