# Supplementary material for: The Information and Communication Technology Maturity Assessment at Primary Health Care Services Across 9 Provinces in Indonesia: Evaluation Study
Source: JMIR Med Inform. 2024 Jul 18;12:e55959. doi: 10.2196/55959 (PMC11269960; doi:10.2196/55959)
Supplement: Multimedia Appendix 2 [file medinform-v12-e55959-s002.docx]

# Multimedia Appendix 1: Questionnaire

# In-depth Interview Guidelines

**Interview objectives:**

Identifying availability, challenges, strengths, weaknesses, and opportunities for improvement (including systems, infrastructure, policies) in the use of information and communication technology (ICT) in the Health Services.

Thank you for agreeing to participate in this interview - we really appreciate your time. This activity is part of the efforts of the Data and Information Center - Ministry of Health, through the Digital Transformation Office (DTO) to explore various information related to ICT in the Health Service in the context of transforming one health data in primary care.

| **Question** |
| --- |
| 1. Opening 2. Name 3. Your position in the Health Service? 4. How long have you worked in your current position? |
| **Information Systems Maturity Assessment**   1. In-depth interview with key points:    1. Human Resource    2. Software and Systems    3. Hardware    4. Infrastructure    5. Challenges and Potential Improvements |

# Maturity Assessment

Level 1: Initial > 1 point

Level 2: Basic > 2 points

Level 3: Good > 3 points

Level 4: Best practice > 4 points

Level 5: Excellent > 5 points

**Description:** The higher the total points obtained, the higher the level of ability (maturity)

**General information**

| Service: |  |
| --- | --- |
| Region (Province, and District/City): |  |
| **MATURITY ASSESSMENT** | |
| **Description of Questionnaire Section** |  |
| Part | 1. Human resources |
|  | 1. Software & system |
|  | 1. Hardware |
|  | 1. Infrastructure |
| Date |  |

| *Part 1: Human Resources* | |
| --- | --- |
| *Question* | *Answer* |
| 1. *Are there adequate personnel for data entry?*   Guide to asking:  *We would like to know whether your agency has the availability of workers for data entry? Do existing personnel help with these tasks?* | 1. Initial > Anyone available can help with data entry 2. Basic > There may be part-time workers 3. Good > Available staff assists with data entry, this can be done according to time availability 4. Best practice > Staff updates data in shifts and on a scheduled basis 5. Excellent > There is a person assigned to focus on data entry tasks   (Probing: Usually in the Health Offices, human resources are limited, so many people do double jobs? Or do data entry staff already have special staff? on a regular basis?) |
| 1. *Are there special personnel dedicated to data entry?*   Guide to asking:  *We would like to know if your agency has a special officer to handle data. This shows management's thoroughness and seriousness in its commitment.* | 1. Initial > Anyone who is available can help 2. Basics > Management shows commitment and interest to support data entry 3. Good > Management assigns dedicated officers to carry out these tasks. 4. Best practice > Management dedicates a team with a good structure to ensure goals are achieved. 5. Excellent > Place highly competent officers in groups and teams, who report to management the results of continuous review and continuous learning.   (Probing: How committed is your agency to providing data entry personnel? |
| 1. *Have the data entry personnel been appropriately trained?*   Guide to asking questions  *We want to know whether the personnel responsible for data handling and entry have received training.* | 1. Initial > Anyone available can help with data entry 2. Basic > Have been trained but may not have sufficient experience or degree 3. Good > Have been trained and have a degree, but may not have enough experience 4. Best practice > Officers who have been trained and have sufficient knowledge, experience and degrees, but may have other duties. 5. Excellent > Dedicate officers who have been trained and have good knowledge and experience.   (Probing: Training is required for data entry workers, what is the intensity of the training? Do data entry workers have appropriate degrees and have appropriate experience?) |
| 1. *Are there special personnel dedicated to data analysis?*   Guide to asking:  *We want to know whether in this workplace there is an officer assigned to handle/manage/analyze data. This shows management's thoroughness and seriousness in its commitment.* | 1. Initial > Anyone who is available can help 2. Basic > Management shows commitment and interest to support data management and analysis 3. Good > Management assigns dedicated officers to carry out these tasks. 4. Best practice > Management dedicates a team with a good structure to ensure goals are achieved. 5. Excellent > Place highly competent officers in groups and teams, who report to management the results of continuous review and continuous learning.   (Probing: What is the agency's commitment to providing data analyst staff? |
| 1. *Have the officers (analysts) been trained appropriately?*   Guide to asking questions  *We want to know whether the person responsible for data analysis has received training.* | 1. Initial > Anyone who is available can help manage and analyze the data 2. Basic > Have been trained but may not have sufficient experience or degree 3. Good > Have been trained and have a degree, but may not have enough experience 4. Best practice > Officers who have been trained and have sufficient knowledge, experience and degrees, but may have other duties. 5. Excellent > Dedicate officers who have been trained and have good knowledge and experience.   (Probing: Training is needed by analysts, what is the intensity of the training? Do analysts have the appropriate degree and experience?) |
| 1. *Are there special officers/IT personnel as information system managers?*   Guide to asking questions  *We want to know whether the officers responsible for the information system have received training.* | 1. Initial > Anyone who is available can help manage the information system 2. Basic > Yes, have been trained but may not have sufficient experience or degree 3. Good > Yes, have been trained and have a degree, but may not have enough experience 4. Best practice > Officers who have been trained and have sufficient knowledge, experience and degrees, but may have other duties. 5. Excellent > Dedicate officers who have been trained and have good knowledge and experience.   Probing: Information Systems management officers have an IT background? What about the training? |
| 1. *Is there a schedule for data entry?*   Guide to asking:  *We want to know about data entry and the schedule. Is there a schedule? Daily data entry with appropriate deadlines? Is it ad hoc?* | 1. Initial > Ad-hoc data entry (When there is time) 2. Basics > There is a delivery deadline schedule, but it is not certain. 3. Good > There is a proper schedule, data is summarized and uploaded manually 4. Best practice > There is a schedule, the data is summarized and the application is ready to help upload 5. Excellent > Upload data automatically, with a good database and regular deadlines. Data is summarized and well documented. There is batch processing.   Probing: What is the mechanism and intensity of data reporting? is it scheduled? |
| 1. *Is there an appropriate knowledge transfer process (entry, information system management, and data analysis) between staff?*   Guide to asking:  *We want to know how knowledge is transferred in this agency. Is there a proper handover or knowledge transfer process between workers? Is there documentation? A proper library of things to do, instructions, or written notes on dos and don'ts?* | 1. Initial > There are no good knowledge sharing sessions, staff have to learn it themselves 2. Basics > There are knowledge sharing sessions, but they are not well documented, only basic instructions 3. Good > There is documentation, there are instructions 4. Best practice > There are knowledge sharing sessions that are held regularly and precisely 5. Excellent > Online knowledge is available to share and learn, officers can carry out their duties well and can ask questions to address the work being done.   Probing: Mutation or rolling between staff is very high, what about the knowledge transfer process between entry officers, analysts and information system managers? Is there a knowledge transfer session? or is it only done via independent study from a guidebook? |
| 1. *What is the knowledge of the staff (for technology and data entry)?*   Guide to asking:  *We want to know whether the officers handling data understand technology? Do they have previous experience? Use technology regularly in their work? Other experiences in the past with partial experience of technology? Can they find more effective ways to complete the data?* | 1. Initial > Basic understanding of technology (internet, applications, etc) 2. Basic > Can use basic applications for data entry 3. Good > Have experience in technology and data entry 4. Best practice > Professional staff who use technology and infrastructure in their daily work 5. Excellent > Fluent in technology and can solve problems in their professional field, can handle and find the right way to improve data entry   Probing: How appropriate is the officer's knowledge and experience regarding information technology? Are you familiar and familiar with existing application systems/technology? |
| 1. *What is the knowledge of the officers (for data analysis applications)?*   Guide to asking:  *We want to know whether the officers who handle the data understand the application of data analysis? Do they have previous experience? Use technology regularly in their work? Other experiences in the past with partial experience of the application/software?* | 1. Initial > Don't know 2. Basic > Can say names, but doesn't understand how to use them 3. Good > Can name and explain basic applications for data analysis, but have never used them 4. Best practice > Can explain basic applications for data analysis, and have used them 5. Excellent > Can explain basic and advanced applications for data analysis, have experience using, and training   Probing: How appropriate is the officer's knowledge and experience regarding data analysis applications? Are you familiar and familiar with existing applications (SPSS, Stata, etc.)? |
| 1. *Is there investment in officers (entry, information system managers and data analysts)?*   Guide to asking:  *We want to learn if there is an investment in officers? Got a budget? Any colleague degrees or skill development? Regular training and exchange of ideas for their future career?*  *The more investment in officers, the better the quality of their work.* | 1. Initial > There is no direct investment in officers in carrying out their duties 2. Basic > Temporary work to perform the task, and no good allocation for future positions 3. Good > There is an officer development program, needs identified 4. Best practice > Good investment in officers, skills, education programs. There's a budget. Encourage certification 5. Excellent > Job rotation, education and training provided, with investment in officers and their future. There's a budget. Exams and career paths for those who excel.   Probing: How is the budget managed to increase human resources capacity (entry, analysts, information system managers)? Is the budget used for human resource development? |

| Part 2: Software and Information Systems | |
| --- | --- |
| Question | Answer |
| 1. *Is there an appropriate and capable information system in your agency?*   Guide to asking:  *We want to know if there is an information system. Is there a shared computer? How do users perform data entry? Data entered from paper form to computer?* | 1. Initial > Only paper and mobile phone 2. Basics > There is a shared computer, data entry from paper to computer 3. OK > Computers, spreadsheets and word documents with data entry on the computer itself 4. Best practice > Data entry on computer, no manual entry 5. Excellent > Cloud computing, no manual entry   Probing: To what extent is the information system utilized in your agency? Are there still manual, semi-manual, or fully online? |
| 1. *Is there technical assistance/support for software & information systems?*   Guide to asking:  Is there any help for users? Software developers still in touch? Is there a contract? If there is a problem, is there someone to contact? | 1. Initial > Ad-hoc assistance (example: Developer vendor, etc.) 2. Basic > Help is available, on a scheduled basis 3. Good > There is maintenance, there is contracted support, on call (help) is available. 4. Best practice > Remote assistance available, 24 hour assistance available 5. Excellent > 24 hour support, help desk available with trouble tickets and escalation procedures   Probing:  Is there a third party technician to resolve system/application/software problems? To what extent is this assistance? |
| 1. *How many software applications are there used in your agency?*   Guide to asking:  The larger the number of applications, the more effort and time required to enter data. We need to know what the effective burden is for data entry officers | 1. Initial > (1-5 apps) 2. Basic > (5-10) 3. Good > (10 – 15) 4. Best practice > (15-20) 5. Excellent > (> 20)   Probing:  How many applications are mandated to be used in your agency? |
| 1. *Of the existing software applications, how many are used effectively and routinely in your agency?*   Guide to asking:  The larger the number of applications, the more effort and time required to enter data. We need to know what the effective burden is for data entry officers | 1. Initial > < 25% 2. Basic > (26 - 50%) 3. Good > (51 – 75%) 4. Best practice > (76 - 100%) 5. Excellent > (100%)   Probing: How many applications are active and currently still routinely used? |
| 1. *Is the data filled in completely in each existing application?*   Guide to asking:  Data completeness is always a problem.  We want to learn why the entered data is only half complete? Are there too many areas that have no purpose?  Is there a difference why one is 100% included and the other is only Partially included? | 1. Initial > Minimum data entry level (30%<) 2. Basic > basic information on personal data or specific fields (30-50%) (decent accuracy) 3. Good > Only fill in what is important, data completeness is half (50 – 75%) (Good level of accuracy) 4. Almost complete > Completeness level > 75% (High level of accuracy) 5. Complete > 100% completeness (high level of accuracy)   Probing:  How is the quality of data filling in each application? |
| 1. *Is there data duplication between the applications used?*   Guide to asking:  We want to learn from the user's perspective whether there is data duplication between the input applications | 1. Too much duplication > (>75%) 2. Some > (50-75%) 3. Slightly > (10-50%) 4. Random duplication > (<10%) 5. No duplication   Probing:  Of the various applications used for data input, to what extent and what is the % duplication rate? |
| 1. *Does the existing information system (application) help facilitate Health Service program activities?*   Guide to asking:  We want to learn from the user's perspective whether all the information systems in your agency make the service process easier? | 1. Absolutely not > The system makes things difficult and burdensome for staff because there are so many problems (many variables, frequent downtime, unstable internet, etc.) 2. No > The system does not make things difficult, but due to limited human resource constraints) 3. Not bad > The system makes work a little easier) 4. Helps make things easier > The system simplifies and speeds up services and reduces staff workload 5. Very easy/smooth > The system simplifies and speeds up services, reduces staff workload, and improves the quality of patient and program services   Probing:  How do existing applications benefit from making daily work easier? What are the obstacles? |

| Part 3: ICT Hardware (Technology, Information and Communication) | |
| --- | --- |
| Question | Answer |
| 1. *Is there an adequate PC/laptop for data entry and analysis?*   Guide to asking:  We want to learn about the hardware where the application is used.  Any PC/laptop?  Is the performance good and stable?  Enough memory to host many applications?  Latest model available? | 1. Initial > Simple/modest PC available (unable to choose due to availability) 2. Basic > Shared PC for multiple purposes 3. Good > Decent hardware to work with (specs may be a bit old). May include a laptop for possible mobile users. 4. Best practice > Dedicated PC/Laptop to do the job with the latest performance specifications. Allows for mobile users. 5. Excellent > Latest model hardware available, allows for mobile users (highest spec laptop/PC)   Probing: What about IT facility support (Computer/Laptop), is each officer given a computer/laptop? |
| 1. *Is there still data that is filled in manually on paper?*   Guide to asking questions  How much data is in a manual formular?  How much power is re-entered into the PC/Laptop?  Is there an automated system to help users? | 1. Initial > Mostly paper based 2. Basic > Some data is entered into the PC/Laptop 3. Good > Most of the data is entered into the PC/Laptop 4. Best practice > Little is paper based, almost all done on PC/Laptop 5. Excellent > There is an automatic system, scanned data from the formular can enter data into the system   Probing: Is the recording system completely online? |
| 1. *Is there a server to process data?*   Guide to asking questions  We want to learn how users process data, and is there an infrastructure? Compiling and processing data requires power and we need to know how the user does it | 1. Initial > Processed via any PC/Laptop 2. Basic > Processed via PC/Laptop with decent specs 3. Good > PC/Laptop with server to process work 4. Best practice > There is a set of special servers to process data on site (in the office/agency) 5. Excellent > Cloud computing to help process data (server not in the office/agency)     Probing: Does it have a dedicated physical server? Or are you already using the cloud? |
| 1. *Is there sufficient storage space to store the data?*   Guide to asking questions  Storage is an issue for data availability  Modern infrastructure can help avoid storage bottlenecks | 1. Initial > available storage space or hard disk 2. Basic > Basic size storage area for storing important data 3. Good > Storage area of decent size and infrastructure, can be upgraded but depends on budget 4. Best practice > Dedicated storage in offices/agencies, stability possible 5. Excellent > Cloud computing for storage   Probing: Data storage using a simple hard disk or do you already have a special physical server? Or are you already using the cloud? |

| Part 4: ICT Infrastructure | |
| --- | --- |
| Question | Answer |
| 1. *Is the internet available?*   Guide to asking:  The internet is a necessity and we need to know the basic internet for each location. What is the condition? | 1. Initial > Internet is available but unstable 2. Basic > Internet is available but not in all areas 3. Good > Internet is available but not high speed 4. Best practice > Internet is available with stability and good performance 5. Excellent > High-speed internet available   Probing: What about internet supply in your agency's work area? How is the connection quality? |
| 1. *Is there a Wifi device available?*   Guide to asking:  Wifi is a device that supports internet access. What is the condition? | 1. Initial > No wifi 2. Basic > There is wifi, but it can't be used 3. Good > There is wifi, it can be used more often, but sometimes it is difficult to use because the *speed capacity* is limited 4. Best practice > There is wifi, can be used more often, and good *speed* 5. Excellent > Has wifi, can be used more often, and is super fast   Probing: What about Wifi device availability? How is the connection quality? |
| 1. *How does internet availability support the data input process?*   Guide to asking:  Support internet availability in your agency, what does it look like? Especially for data input and analysis needs | 1. Initial > Use more personal data packages 2. Basic > Personal data and agency wifi 3. Good > Agency Wifi, but connection is slow 4. Best practice > Agency Wifi, and smooth 5. Excellent > Agency Wifi, smooth, and support for additional data package funds   Probing: What is the mechanism for using internet packages in the data input process? Does anyone still use personal data packages other than WiFi? |
| 1. *Is electricity and power available?*   Guide to asking:  We want to study the availability of electricity and power in this area.  Is there electricity and a power supply system?  Is there electricity and power backup?  Electrical safety and any power available. | 1. Initial > Network infrastructure is available with limited specifications (cables, routers, switches, etc.). There is no reliable backup power supply. 2. Basics > Electricity and basic power supplies 3. Good > Electricity and power supply are good 4. Best practice > Electricity and stable power supply with good and reliable performance. There is electricity and power backup 5. Excellent > Electricity and stable power supply with the Excellent and most reliable performance. There is security of electricity and power supply.   Probing: How is the electricity condition in your agency's work area? Are there frequent blackouts? Does your agency have its own generator? |
| 1. *Is physical infrastructure available?*   Guide to asking:  Physical infrastructure is important to ensure good performance and special attention is paid to supporting ICT. Is there a dedicated space for networking?  Is there a data center available? | 1. Initial > No dedicated network or data center 2. Basic > Combined area for network and data center 3. Good > There is dedicated space for networks and data centers 4. Best practice > There's room for both a separate network and data center, with good specs 5. Excellent > Excellent-in-class networking and data centers in the cloud. Advanced infrastructure.   Probing: Is there a dedicated data center and server/network building? |

| Part 5: Challenges for HR, Information Systems, ICT Hardware and Infrastructure | |
| --- | --- |
| Question | Answer |
| 1. *Human resources challenges?* | Free Text    Probing: HR is an important asset in every agency, what are the HR challenges in your agency in using Information Systems? |
| 1. *Information System (Application) Challenges?* | Free Text  Probing: Information systems sometimes have shortcomings, so what problems has your agency often encountered regarding software/applications? |
| 1. *Hardware Challenges* | Free Text  Probing: Hardware support is very important for running information system software. So far, what problems has your agency often encountered related to hardware? Is a laptop/computer enough? Competent Wifi device? |
| 1. *Infrastructure Challenges* | Free Text  Probing: What about other supporting infrastructure? For example, a server room |
| 1. *Input for digital transformation* | Free Text  Probing: In the context of digital transformation of the Primary Health Care recording system, what is your input? |
